# Enhanced light collection in fluorescence microscopy using self-assembled micro-reflectors

**DOI:** 10.1038/srep10999

**Published:** 2015-06-17

**Authors:** Zoltán Göröcs, Euan McLeod, Aydogan Ozcan

**Affiliations:** 1 Department of Electrical Engineering, University of California Los Angeles (UCLA), CA 90095, USA; 2 Department of Bioengineering, University of California Los Angeles (UCLA), CA 90095, USA; 3 California NanoSystems Institute (CNSI), University of California Los Angeles (UCLA), CA 90095, USA

## Abstract

In fluorescence microscopy, the signal-to-noise ratio (SNR) of the optical system is directly linked to the numerical aperture (NA) of the microscope objective, which creates detection challenges for low-NA, wide-field and high-throughput imaging systems. Here we demonstrate a method to increase the light collection efficiency from micron-scale fluorescent objects using self-assembled vapor-condensed polyethylene glycol droplets, which act as micro-reflectors for fluorescent light. Around each fluorescent particle, a liquid meniscus is formed that increases the excitation efficiency and redirects part of the laterally-emitted fluorescent light towards the detector due to internal reflections at the liquid-air interface of the meniscus. The three-dimensional shape of this micro-reflector can be tuned as a function of time, vapor temperature, and substrate contact angle, providing us optimized SNR performance for fluorescent detection. Based on these self-assembled micro-reflectors, we experimentally demonstrate ~2.5-3 fold enhancement of the fluorescent signal from 2-10 **μ**m sized particles. A theoretical explanation of the formation rate and shapes of these micro-reflectors is presented, along with a ray tracing model of their optical performance. This method can be used as a sample preparation technique for consumer electronics-based microscopy and sensing tools, thus increasing the sensitivity of low-NA systems that image fluorescent micro-objects.

Fluorescence microscopy is one of the most commonly used methods in biomedical assays due to its ability to provide specificity and sensitivity for various detection and sensing applications^1^,^2^,^3^,^4^,^5^,^6^. In recent years, researchers have developed portable fluorescent microscopes to address various global health needs through the use of consumer electronic components, e.g. cellphones^7^,^8^,^9^ or flatbed scanners^10^,^11^. While these devices tend to be low-cost and typically provide high throughput due to their low numerical aperture (NA), large field-of-view optics, these advantages also come at the expense of reduced sensitivity to small fluorescent objects.

Several methods have been devised to effectively increase the detected fluorescent signal from micro-objects. For example, photonic crystals have been designed to utilize strong coherent scattering effects and high intensity near fields to enhance the fluorescent emission^12^,^13^. Plasmonic nanostructures have been extensively used to enhance the spontaneous emission from molecules near a metallic surface^14^,^15^,^16^. These methods are based on the overlap between the emission spectrum of the fluorophore and the surface plasmon mode frequency^17^. Enhancement of fluorescent detection by redirecting the emitted light towards the optical system, thus increasing the collection NA of the lens has also been achieved for single molecule fluorescent detection using solid immersion lenses positioned between the target object and the detector^18^,^19^,^20^,^21^. Recently, a fluorescent enhancement method based on manufacturing multiscale wrinkled silica structures on shrink-wrap film has been used to increase the signal-to-noise ratio for DNA microarrays^22^. Self-assembly of colloidal particles to act as an array of large NA lenses and increase the detected fluorescent intensity has also been successfully used on surface-immobilized single fluorophores^23^. Most of these methods deal with single fluorescent molecules and require the molecules to be approximately in the optical near field of the enhancing surface.

Here we present a simple alternative method to increase the fluorescent sensitivity of optical imaging systems by depositing a liquid micro-reflector around the target sample through vapor condensation (Fig. 1a). Vapor deposition and droplet growth on transparent surfaces have intrigued physicists for more than a century^24^. Recently, self-assembly of liquid nano-lenses around nano-particles has been used for enhancing the signal-to-noise ratio in holographic on-chip microscopy, enabling label-free detection of sub-40 nm particles^25^,^26^. For this purpose, polyethylene glycol (PEG) is an ideal material for creating evaporation-based self-assembled optical components as it is non-toxic, and has a sufficiently high vapor pressure to be able to evaporate at moderate temperatures (e.g. ~100 °C), but low enough to be stable for *several hours at room temperature*. Furthermore, evaporation based self-assembled PEG nanolenses have been successfully integrated with cost-effective and field-portable holographic imaging devices^27^. The shape of the PEG meniscus depends on the contact angles of the polymer on the sample substrate and on the particle. Unlike its prior uses in digital holography for label-free bright-field imaging, where the enhancement of the scattered signal is achieved by depositing liquid nanolenses with a low surface contact angle, here we engineer these self-assembled vapor-condensed lenses to improve the sensitivity of *fluorescence* microscopy by using larger substrate contact angles to shape the meniscus as a *reflector* that increases the excitation efficiency and redirects laterally-emitted fluorescent light away from the sides and towards a detector located below the micro-reflector, thereby strongly enhancing the measured fluorescence signal for each micro-particle (see Fig. 1).

These self-assembled micro-reflectors are particularly effective in large-FOV, low-NA imaging systems where laterally-emitted light would not normally be collected. To quantify the improvement due to the reflectors, we used *in situ* low-NA (~0.1) fluorescence microscopy to monitor the change in fluorescent signal levels from individual beads during the self-assembly of the micro-reflectors as the PEG vapor condenses. Although we used a scientific-grade fluorescence microscope in this study, similar results can be expected from other, more cost-effective fluorescence imaging platforms with a similar NA^10^,^28^. We studied spherical beads because (i) they represent a common fluorescence standard and (ii) they provide simpler comparison between experiment and theory based on axisymmetric geometries. We found that when optimized, PEG reflectors can achieve ~2.5-3 fold enhancement of the fluorescent signal from individual micro-particles. This optimization required moderately large contact angles relative to the substrate, which we achieved using a polymer based microscope coverslip with a surface contact angle of ~30°. Furthermore, we compared our experimental results to numerical models implemented using ray tracing simulations, which helped us better understand how the size and shape of the liquid micro-reflectors enhance the collected fluorescent light through internal reflections at the air-PEG interface.

## Materials and Methods

To visualize the micro-reflector formation *in situ* and its effect on the detected fluorescence intensity, we combined a custom-designed vaporization chamber with a standard upright fluorescence microscope.

### Fluorescent microsphere sample preparation

A Rinzl plastic coverslip (Electron Microscopy Solutions, 72261-22) is cleaned using deionized water. Fluorescent polystyrene microspheres with 10 μm (Magsphere, PSF-010UM), 5 μm (Magsphere, PSF-005UM), and 2 μm (Magsphere, PSF-002UM) sizes are diluted in isopropanol with a 1:1000 ratio. 2 μl of this solution is pipetted onto the polymer (Rinzl plastic coverslips, Electron Microscopy Sciences) coverslip and set aside for 15 minutes to let the isopropanol completely evaporate.

### Evaporation chamber construction

To create the micro reflectors around the fluorescent beads, a custom-made 3D-printed evaporation chamber was designed. An insulated flexible heater (Kapton KHLV-101/(10)-P) is taped into the bottom of a 3D printed tray. Approximately 0.5 ml of PEG 300 polymer is placed onto the heater. A 3D printed cone is used to cover the heater and to funnel the vapor to the sample. The speed of the evaporation, and thus the speed of the liquid reflector growth can be increased by increasing the voltage across the heater. In our experiments we used voltages between 13 – 17 V corresponding to a heater temperature of 100 °C – 120 °C.

### Microscope setup

**Figure 1:**
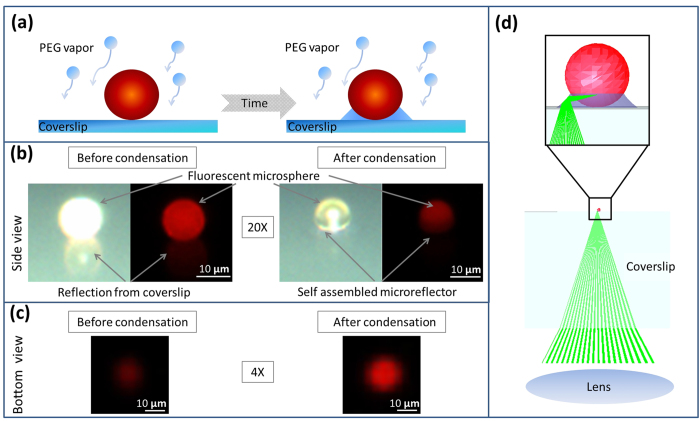
Self assembled micro-reflector formation. (**a**) As the liquid condenses around the fluorescent particle, a meniscus forms over time. (**b**) Side view images of the same 10 μm fluorescent particle before and after the meniscus formation using the same illumination and camera settings. Both reflected bright field and fluorescent 20× microscope images are shown. Note the absence of the reflection from the coverslip holding the sample, as this light gets redirected downward into the coverslip as indicated in (**d**). Also note that the condensation process forms a thin liquid layer on the fluorescent particle, reducing its surface roughness, thus lowering the scattered light intensity from the bead and confining some of the fluorescent emission within the bead body. PEG is highly transparent, thus the visibility of the meniscus is low. (**c**) Bottom view of the same particle before and after the meniscus formation using a 4× microscope objective, which qualitatively shows the increase of the detected fluorescent light. The measurements were taken using the same illumination and camera settings. (**d**) Fluorescent light emitted by the bead outside the acceptance angle of the optical system will be redirected towards the detector due to reflections on the surface of the meniscus.

The evaporation chamber is placed into the condenser lens port of an Olympus BX-51 microscope. A 3D printed holder is used to hold the Rinzl substrate with the sample being upside down facing the evaporation chamber. A 4× Olympus microscope objective (NA = 0.13) in conjunction with a monochrome video camera (QImaging QIClick-F-M-12) at the video port of the microscope is used to measure the fluorescent signal change during the micro-reflector formation by capturing a raw image in every 2 seconds. For better visualization a color camera (Qimaging Retiga 2000R) was used to acquire the images shown in Fig. 1.

### Data analysis

The created image stack is analyzed frame by frame. The fluorescent microparticles are detected, localized, and segmented at each frame. Clusters of beads are automatically rejected based on their occupied area on the first frame. The measured intensity of a single bead is the total intensity of the segmented area, thus we take into account the intensity redirected by the reflector which arrives next to the bead increasing its lateral size. During the evaporation process the sample holder suffers from heat expansion, which slightly shifts the sample. The segmented and selected single beads are automatically located and tracked frame by frame to negate this effect as well as to take into account the few particles that move due to surface forces caused by coalescing droplets.

## Results and Discussion

We tested our approach on fluorescent microparticles with sizes between 2 and 10 micrometers to study the effect of the particle size on meniscus formation and resulting signal enhancement. In each experiment, several hundred microbeads were deposited onto a Rinzl microscope coverslip and were imaged by a 4× microscope objective (NA = 0.13). Larger particles require more material deposited around them to form the ideal reflector shape, thus for a given vapor flux, micro-reflectors form more quickly around smaller particles than larger particles. The speed of the vapor deposition and the reflector formation also depends on the temperature of the heater inside the PEG reservoir, which we adjusted to be between 100 °C and 120 °C to keep the reflector formation on a similar timescale for different bead sizes. Note that these temperatures are well below the boiling point of PEG (molecular weight: 300 Da), which is >220 °C. Also note that the maximum temperature of the sample during the entire vapor deposition process is much cooler than the reservoir, and has been measured to be approximately 37 °C. While these PEG micro-reflectors were forming around each particle of interest, the fluorescent intensity of the particles was sampled at 2 second intervals using a monochrome camera attached to the same microscope.

**Figure 2:**
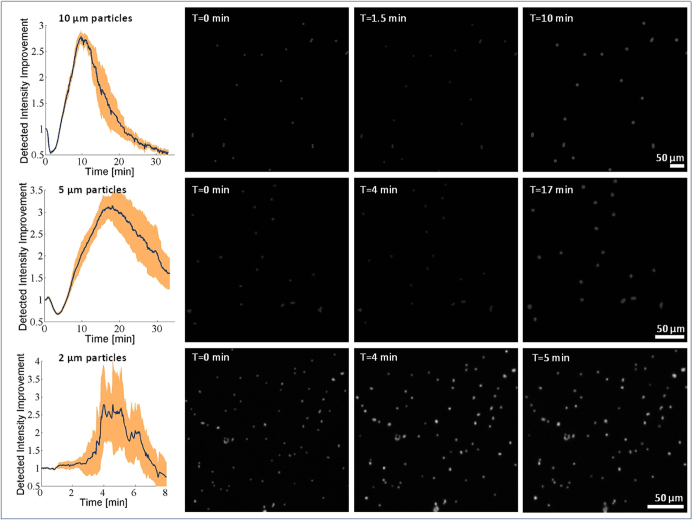
Measured fluorescent intensity enhancement during the PEG micro-reflector formation process for 10 μm, 5 μm, and 2 μm fluorescent beads, respectively. Fluorescent microscope (4×; NA = 0.13) images are shown at different time points. The width of the shaded orange region for each curve is equal to twice its standard deviation.

The results of our imaging experiments are summarized in Fig. 2, where for all the bead sizes a maximum intensity increase of 2.5-3 fold with respect to the initial state (2^nd^ column) was observed once the optimum micro-reflector shape (last column) had been achieved. Interestingly, for 10 μm and 5 μm particles, the normalized fluorescent intensity plots (Fig. 2, left column) show an initial drop in the relative intensity to 0.5 and 0.7, respectively, before it starts to increase. In the case of 2 μm particles, however, the initial drop in intensity was not observed (Fig. 2, 3^rd^ row). As we discuss in more detail below, we attribute these initial decreases in the relative fluorescent intensity of the micro-bead to the PEG condensation that effectively smoothens the bead surface, causing light to be better trapped within the micro-particle due to the whispering gallery modes^29^. However, at later times, the fluorescent signal enhancement resulting from the micro-reflector dominates the loss from the smoothening effect, resulting in a net enhancement in the collected light (Fig. 2, rightmost column).

To quantitatively understand these observations and the mechanism behind the increased fluorescent collection efficiency observed using the vapor-condensed self-assembled micro-reflectors, we created a mathematical model of the condensation of liquid around a fluorescent particle, and its resulting optical properties for fluorescent emitters. The rate of formation of the micro-reflector and its evolving shape during formation are analytically calculated by computing the vapor flux for a given PEG reservoir temperature and solving the Young-Laplace equation^26^,^30^,^31^,^32^. This reflector size and shape as a function of time are then used as input to a ray tracing algorithm, which calculates the fluorescent intensity variations as a function of vapor condensation and micro-reflector self-assembly.

The modeling approach discussed here for the rate of formation and shape evolution of the micro-reflectors has been adapted for the drop-wise condensation of micro-reflectors observed on low surface energy substrates, as opposed to the previously-published film-wise condensation of nanolenses on high surface energy substrates^26^. Based on the observation of drop-wise condensation, we assume that the adsorbed microspheres act as nucleation sites. In particular, nucleation can occur on the surface of the microspheres (which are not completely smooth) as well as the triple-junction between the particle, the substrate, and the vapor. Once a critical nucleus is formed, capillary flow will rapidly cause the condensate to accumulate at the triple-junction. Subsequent condensation will then occur at the growing liquid-vapor interface that defines the liquid meniscus shape.

The dynamics of the first stages of condensation are determined by the time required for a critical nucleus of PEG molecules to condense around a bead. Based on numerical estimates and direct experimental observation, we find this time to be very fast (<1 s) compared to the subsequent growth process (minutes). After the formation of the critical nucleus, the volumetric growth rate of the micro-reflector is proportional to the molecular flux in the vapor and the liquid-vapor surface area:


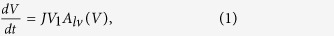


where *J* is the molecular flux in the vapor, *V*_1_ = 4.15 × 10^−28^ m^3^ is the volume of a single PEG molecule in liquid phase, and *A*_*lv*_(*V*) is the liquid-vapor surface area as a function of micro-reflector volume. To simplify the modeling of the condensation procedure, we assume steady-state, neglect the initial transient heating period, and assume that the chamber contains PEG vapor at constant density, resulting in a constant flux *J*, which can be calculated from the Maxwell-Boltzmann distribution^33^:


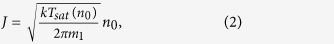


where *k* is Boltzmann’s constant, *m*_1_ = 4.69 × 10^−25^ kg is the mass of a single PEG molecule (assumed to have 6 monomer units so that the molecular weight is ~300 Da), *n*_0_ is the number density of molecules in the vapor, and *T*_*sat*_(*n*_0_) is the saturated vapor temperature at ambient pressure:


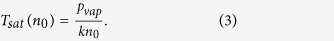


Here, *P*_*vap*_ is the partial pressure of the saturated vapor, which we have extrapolated from empirical vapor pressure curves of different molecular weights of polyethylene glycol^34^:


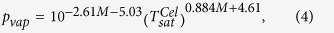


where 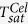 is the saturated vapor temperature in Celsius, and *M* is the number of monomers in the polymer chain.

The initial condition at *t ≈* 0 for the differential equation (1) can be taken as the volume of a liquid monolayer occupying the triple-junction:


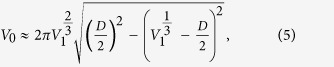


where *D* is the diameter of the microsphere serving as the nucleus.

**Figure 3:**
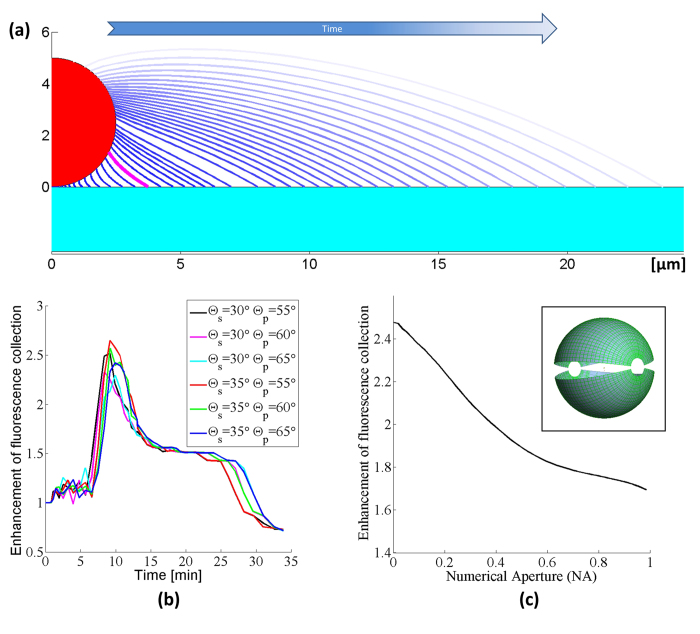
(a) Simulated reflector shapes with contact angles Θ_s_ = 30°, Θ_p_ = 60°. The size of the liquid meniscus increases with time as the vapor is deposited. The meniscus corresponding to the maximum enhancement of fluorescence collection is drawn in magenta. (**b**) The predicted enhancement factors resulting from ray tracing simulations for various substrate (Θ_s_) and particle (Θ_p_) contact angles. The set of contact angles best matching the experimental case is shown in magenta. (**c**) Calculated emitted intensity improvement of the magenta-colored reflector shape vs. the numerical aperture of the optical system (contact angles: Θ_s_ = 30° ; Θ_p_ = 60°) using a spherical 5 μm fluorescent particle. The light is mainly redirected towards the optical axis of the imaging system. During the non-sequential ray tracing simulations, 2000 emitters are placed randomly into the fluorescent sphere with uniform distribution. Every emitter releases 2000 rays into the full 4π solid angle with uniform distribution for a total starting ray count of 10 million. The simulation assumes a perfect spherical particle before the micro-reflector deposition, thus ~50% of the emission is initially confined inside the particle due to the total internal reflection on the particle surface. Inset shows the detectors located ~10 cm above and below the bead. The two hemispheres detect the emitted intensity into angles between −80° to 80° with an angular bin size of 1°.

In addition to the molecular flux *J*, the solution to Equation (1) also requires the relationship between the micro-reflector volume *V* and its liquid-vapor surface area *A*_*lv*_. This relationship depends on micro-reflector shape, which can be modeled using the Young-Laplace equation^31^,


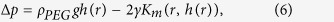


where Δ*p* is the pressure difference across the air-liquid interface, *ρ*_*PEG*_ is the liquid PEG density, *g* is the acceleration due to gravity, *h*(*r*) is the height of the liquid-air interface, *γ* is the PEG surface tension, and *K*_*m*_ is the local mean curvature of the interface. Equation (6) becomes a nonlinear second-order ordinary differential equation when the explicit expression^26^ for *K*_*m*_ is substituted. We solve this equation numerically using an initial-value approach. We compute the interface shape for a set of different contact points at the bead, all having a given contact angle with the bead of *θ*_*p*_, lying within the range 55° to 65°, based on the side-view optical micrograph shown in Fig. 1B. Out of the set of interface shapes corresponding to different contact points on the bead, we select the interface shape that provides a close match (within 0.3°) to the desired contact angle at the substrate *θ*_*s*_, which is known from the side-view optical micrograph in Fig. 1b to lie within the range 30° to 35°. We perform this computation for a large range of Δ*p* values, which correspond to different micro-reflector volumes *V*. These computations give us the shape of the micro-reflector at each stage of growth in volume. The rate at which the micro-reflector grows and adopts these shapes is then computed via Equation (1), the results of which are summarized in Fig. 3a.

After computing the expected size and shape of the micro-reflectors (Fig. 3a), we used a non-sequential ray tracing algorithm to calculate the effective fluorescent intensity enhancement. Our ray tracing model uses an ideal sphere with a diameter of 5 μm made out of polystyrene (n = 1.61), although the simulation results for relative enhancement of fluorescent light collection should be independent of the sphere diameter as long as we are in a size regime where ray tracing is valid (i.e., significantly larger than the wavelength). In accordance with the experimental conditions, we model the sphere and micro-reflector as resting on a Rinzl coverslip (n = 1.5) with a thickness of 180 μm. To model the fluorescence emission from this sphere, 2000 emitters are placed randomly with a uniform distribution into the sphere. Every emitter is a starting location for 5000 rays that launch into the full 4π solid angle around the emitter with uniform distribution, yielding a total starting ray count of 2000 × 5000 = 10 million for each run. Our simulation accurately calculates the relative intensity of each ray as it splits and creates subsequent reflected and transmitted rays every time the ray intersects a surface. Therefore, the rays reaching either of the detectors will carry their relative intensity information, including the partial losses due to subsequent reflections. In this ray tracing model, we take into account the reflector formation by calculating 70 reflector shapes with increasing amounts of liquid in them (see Fig. 3a), representing different points in time during the vapor condensation process. The ray tracing is done for all of these reflector shapes sequentially. At 10 cm distance below and above the sample plane, we placed two hemispheres as detectors, detecting the emitted intensity into angles between −80° to 80° in both directions using an angular bin size of 1° (see Fig. 3 inset). Using this ray tracing framework, the total intensity of the fluorescent light emitted into the acceptance cone of the 4× microscope objective (NA = 0.13) used in our measurement setup is also calculated. Similar to Fig. 2, the detected intensity for various reflector shapes is normalized by the intensity calculated without any micro-reflector around the particle. To take into account the difficulty of obtaining the exact contact angles from the side-view microscope image (Fig. 1b), we also investigated the effect of minor contact angle changes on the calculated intensity improvement curves and found the variations to be small as illustrated in Fig. 3b.

In the initial comparison of our experimental results of fluorescence intensity enhancement (Fig. 2, left column) to our theoretical model (Fig. 3b), we find good qualitative agreement; however there are also some interesting differences. Both sets of curves show clear maxima in intensity, with values that agree reasonably well (~3 times enhancement). On the other hand, before the enhancement of the micro-reflectors sets in, the experimental curves show an initial drop in intensity, which is not seen in our model predictions.

A closer examination of the conceptual differences between our model and experiments can elucidate some possible reasons for this difference observed at early times (≤3 min). First, our model uses a perfect sphere to mimic each fluorescent particle, which causes ~50% of the light to get trapped in the particle due to total internal reflection on the particle surface (e.g., in the form of whispering gallery modes^29^). In contrast, the shape of a real fluorescent microbead is not perfectly spherical and typically has a surface roughness of about 1% of the particle radius^35^,^36^,^37^,^38^. This constitutes a more diffuse particle surface due to increased scattering, which inhibits light trapping and has a higher fluorescent output compared to a perfect sphere. However, during the deposition of the PEG onto the sample, the liquid also forms a smooth layer on the particle that effectively makes the bead more spherical, with reduced surface roughness. This initially reduces the fluorescent output from each bead through light confinement in the form of whispering gallery modes (see Fig. 1b), and can potentially explain the initial experimental drop-off in the collected fluorescent intensity as illustrated in the left column of Fig. 2. As the vapor continues to condense around the fluorescent particle, however, the reflector starts to self-assemble and the emitted fluorescent light from each particle will be redirected towards the detector, thereby significantly increasing the fluorescent intensity measured, even beyond the initial intensity boost provided by the particle’s native surface roughness.

**Figure 4:**
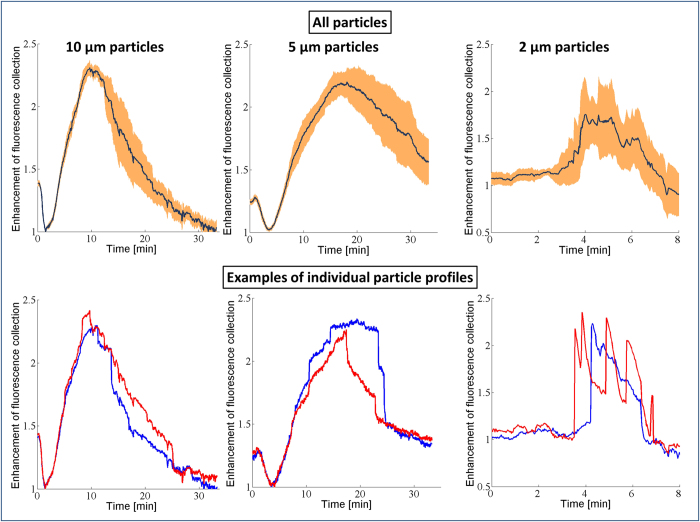
Enhancement of fluorescence collection during the PEG micro-reflector formation process for 10 μm, 5 μm, and 2 μm fluorescent beads, respectively. In order to match our simulations, the emission data was normalized to the first minimum on the curve, corresponding to a smoothened fluorescent bead (through PEG deposition), and the square root of the intensity was taken to account for the increased excitation caused by the micro-reflector. Regardless of the bead size, the peak values for the enhancement of fluorescence collection are ~2.3-2.4, agreeing very well with our model results shown in Fig. 3b. For 2 μm fluorescent particles, several local maxima in time (i.e., blinking) were observed.

A second conceptual difference between our model and experiments is that the optical model only calculates the fluorescent light emitted from the particle and does not consider possible enhancement of the excitation light due to the micro-reflector. When we compensate for these two conceptual differences between our model and experiments, we can find even better agreement between the experimental results and numerical simulations. To compensate for the particle smoothening effect, we rescale the measurement results to take the minimum point of the intensity curve as the new normalization factor, instead of the initial intensity point, which we used in Fig. 2. This change effectively makes our reference point that of a perfect sphere (after PEG deposition) instead of a rough native sphere. In addition to this, we also aimed to isolate the effects of increased excitation due to the micro-reflector shape. In our ray tracing calculations, a substantial amount of the enhancement of the fluorescent collection originates from the light rays that get reflected from the meniscus surface and leave the sample plane adjacent to the bead (see Fig. 1d). Since the same lens is used for both excitation and collection of the fluorescent emission, by using a geometrical optics approximation we can treat the light rays as reversible, and for a given reflector shape we can consider the excitation enhancement factor due to the micro-reflector to be approximately equal to the enhancement factor of the fluorescence collection. In other words, the physical source of the excitation enhancement can be considered to be the internal reflections of the excitation rays at the micro-reflector surface, re-directing them toward the embedded body of fluorophores within a given particle. By using this assumption, we can take the square root of the experimentally-measured intensity curves to separate the component of the enhancement that is due to increases in the collection efficiency alone, apart from the component that is due to excitation enhancement. Figure 4 shows our experimental data after making these two adjustments: in both the numerical model results for fluorescence collection (Fig. 3b) and the normalized/adjusted experimental results (Fig. 4), the peak values for the enhancement of fluorescence collection are ~2.3-2.4×, providing a decent agreement to each other. Here, we should also emphasize that the overall fluorescence enhancement peak factor due to these self-assembled micro-reflectors is larger as shown in Fig. 2 since it also includes an additional source of improvement due to enhanced excitation.

Through Fig. 4, we can also revisit another interesting feature of our experimental results that is not found in the model: the smallest (2 μm) beads exhibit a pronounced blinking effect (Fig. 4, bottom row). We attribute this effect to the spatially and temporally stochastic nature of the vapor condensation process. For smaller particles, these stochastic effects are more apparent due to the reduced areas of interaction between the bead, reflector, and substrate. As a result, for the 2 μm beads, the growth curves show more fluctuations than are seen for the larger beads. We expect this relative blinking behavior to be indicative of fluctuations in the size of the reflector, most likely due to coalescence between the reflector and other non-specific droplets that nucleate at defects on the polymer substrate (see Supplementary Fig. S1 for further information). Nonetheless, the maximum enhancement of the collected fluorescent light in our experiments also agrees very well with our model predictions for these small beads, along with the larger ones as illustrated in Figs. 3b and 4. The exact speed and dynamics of the reflector formation in our model assume individual particles with a single liquid meniscus growing around them, and ignore droplets coalescing with each other thus the exact shape of the theoretically-predicted curves only approximate the measured results.

Our optical modelling and simulation results can also be utilized to calculate the theoretical improvement of the emitted fluorescent intensity as a function of the NA of the photon collection or imaging system (see Fig. 3c), which is especially helpful in evaluating the performance of this approach when applied to other imaging systems. Note that for an NA of 0.13 (the NA of our experimental set-up), the predicted intensity improvement plotted in Fig. 3c agrees very well with that shown in Fig. 4 (~2.35 times enhancement). Figure 3c also shows that even for high-NA systems, we expect that this technique will work favorably to enhance the collection of fluorescent signals, albeit with slightly reduced enhancement factors.

Note that the above results use spherical particles both in the simulation model and in the experiment to allow the use of the axisymmetric Young-Laplace equation, and because fluorescent microspheres are a common fluorescence standard. The same method can be generalized to non-spherical objects as well as clusters of micro-objects, however we expect a different fluorescent enhancement factor for such particles due to their shape, which will affect the self-assembled reflector shape and performance; for example refer to Supplementary Fig. S2 for our experimental results on micro-reflector formation around particle clusters, providing a similar fluorescent signal enhancement as predicted by our model for single particles.

## Conclusion

In conclusion, we have developed a method to fabricate liquid micro-reflectors around micron-scale fluorescent particles to enhance the excitation and fluorescence collection efficiencies when imaging such particles in a microscope system. The fluorescence collection enhancement is due to the redirection of laterally-emitted light into the collection aperture of the system; a similar argument also applies for excitation enhancement due to internal reflections of the excitation rays toward the embedded fluorophores within the particle. To understand and optimize these reflectors, we have developed a numerical model for their size, shape, and impact on the fluorescence signal as a function of time. When appropriately applied, this model agrees very well with the experimental results. While the approach provides enhancement at all levels of NA, we find it most useful for large-FOV, low-NA systems, where the initial SNR may be quite low and an enhancement factor of 2.5-3× provided by these self-assembled and room temperature stable micro-reflectors is significant enough to make small fluorescent particles visible and detectable in such systems. Hence the proposed method could be especially useful for relatively low NA microscopic imaging, sensing and diagnostic systems based on cellphones or other consumer electronics devices^11^,^39^.

## Additional Information

**How to cite this article**: Göröcs, Z. *et al.* Enhanced light collection in fluorescence microscopy using self-assembled micro-reflectors. *Sci. Rep.* **5**, 10999; doi: 10.1038/srep10999 (2015).

## Supplementary Material

Supplementary Information
