# Supplementary material for: Enhanced light collection in fluorescence microscopy using self-assembled micro-reflectors
Source: Sci Rep. 2015 Jun 17;5:10999. doi: 10.1038/srep10999 (PMC4470325; doi:10.1038/srep10999)
Supplement: Supplementary Information [file srep10999-s1.pdf]

Supplementary Information

Enhanced light collection in fluorescence microscopy using self-assembled micro-reflectors

Authors: Zoltán Göröcs[1§], Euan McLeod[1§], and Aydogan Ozcan[1,2,3]*

[1]Department of Electrical Engineering, University of California Los Angeles (UCLA), CA 90095, USA

[2]Department of Bioengineering, University of California Los Angeles (UCLA), CA 90095, USA.

[3]California NanoSystems Institute (CNSI), University of California Los Angeles (UCLA), CA 90095, USA

[*]Corresponding Author: Prof. Aydogan Ozcan

E-mail: ozcan@ucla.edu   Web: http://www.innovate.ee.ucla.edu/

Micro-reflector formation around 2 μm particles.

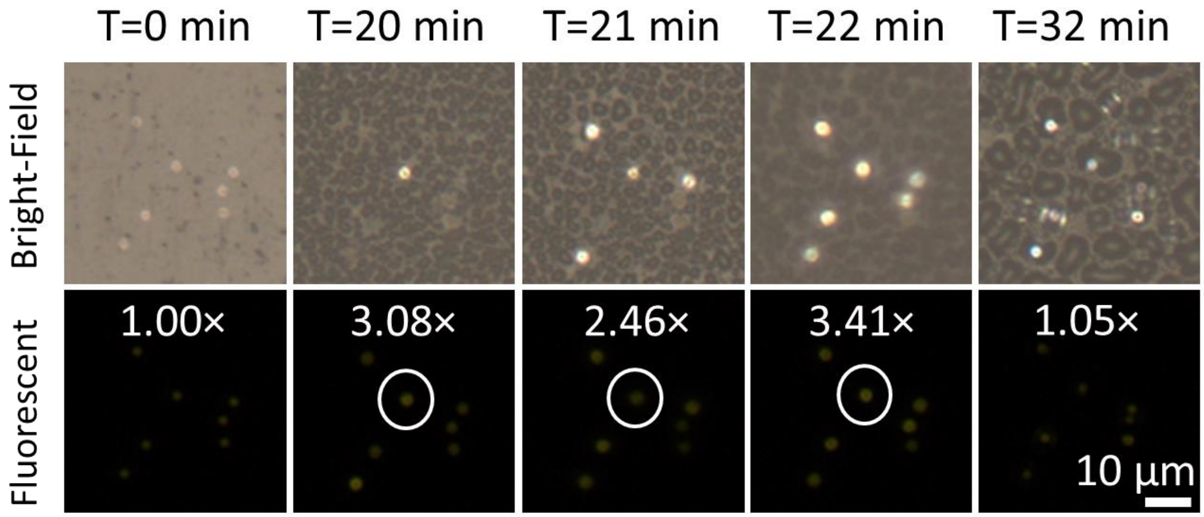

Supplementary Figure S1: Time-lapse reflected bright-field (20× objective; NA=0.5) and fluorescent microscope images of the formation of the liquid micro-reflectors around 2μm fluorescent particles. The encircled particle in the fluorescent images exhibits the "blinking effect". The particle's relative intensity increase for each corresponding fluorescent image is also indicated on the image. We attribute this effect to the stochastic interactions of the liquid reflector with other non-specific droplets on the substrate. Smaller particles are more susceptible to this effect as the liquid meniscus volume becomes comparable to the average size of the nonspecific droplets.

Micro-reflector formation around particle clusters.

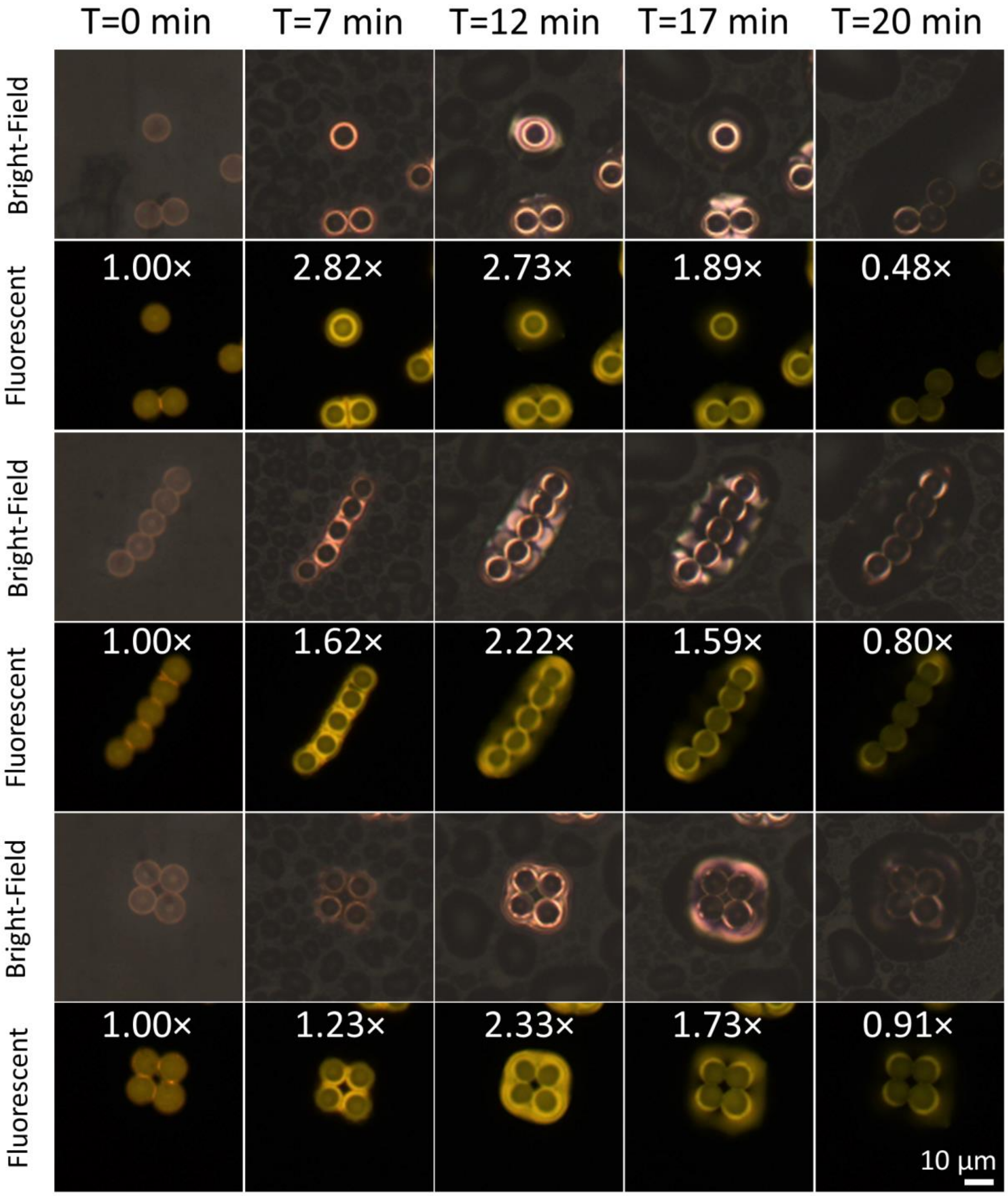

Supplementary Figure S2: Reflected bright-field (20× objective; NA=0.5) and corresponding fluorescent images of 10μm fluorescent particles during micro-reflector formation showing particle aggregates at different configurations. The detected intensity improvement created by the liquid micro-reflectors shows time and performance variation depending on the configuration of particles, but shows a very similar performance to that predicted by our model for single particles. The improvement is calculated by integrating the total light gathered from each particle configuration and dividing it by the intensity measured at T=0.
